# Tissue apoptosis in mice infected with *Leptospira interrogans* serovar Icterohaemorrhagiae

**DOI:** 10.1186/s40409-015-0022-y

**Published:** 2015-07-28

**Authors:** Márcia Marinho, Cilene Vidovix Táparo, Itamar S. Oliveira-Júnior, Silvia Helena Venturoli Perri, Tereza Cristina Cardoso

**Affiliations:** Department of Support, Production and Animal Health, Veterinary Medicine School, São Paulo State University (UNESP – Univ Estadual Paulista), Araçatuba, São Paulo Brazil; Department of Inflammatory Mediators, Anesthesiology, Pain and Intensive Care, UNIFESP, São Paulo, São Paulo Brazil

**Keywords:** Leptospirosis, Caspases, Programmed cell death, Mice, Inflammatory response

## Abstract

**Background:**

This investigation aimed to evaluate the occurrence of some apoptotic features induced by *Leptospira interrogans* serovar Icterohaemorrhagiae infection in young BALB/c mice during 2, 4, 7, 10, 14 and 21 days post-infection (dpi).

**Methods:**

The animals were euthanized and lung, liver and kidneys were harvested to histopathology analysis and immunohistochemistry to caspase-3 antigen detection was performed.

**Results:**

Chromatin condensation in kidney and liver tissues, but not in lung tissue, was observed. Caspase-3 reactive cells, mainly characterized as renal epithelial cells, were detected in the days 14 and 21 at high levels when compared to days 2, 4 and 7 (p = 0.025; p < 0.05). Lung sections revealed caspase-3 labeled alveolar cells in 10 and 14 days post-infection was higher than observed at 7 days (p = 0.0497; p < 0.05). Liver sections demonstrated reactive cells at a highest level at 14 and 21 days post-infection when comparison to 2, 4, 7 and 10 days (p = 0.0069; p < 0.05).

**Conclusions:**

Our results suggest that infection of *L. interrogans* induce in kidney, liver and lung an activation of apoptosis mediated by caspase-3 dependent pathway in later phases of infectious process.

## Background

Leptospirosis is an important zooanthroponotic disease spread worldwide which infection is recognized as a re-emergent disease [1]. The spirochetes of the genus *Leptospira* are responsible for human and animal leptospirosis characterized as mild febrile illness to severe multiorgan failure, especially pulmonary hemorrhage and renal failure [2]. Pathogenic leptospires are highly motile and invasive spirochetes that have the capacity to survive and grow in tissue by escaping natural defense mechanisms [3]. The disease is transmitted to humans by direct or indirect exposure to contaminated urine from mammalian reservoir hosts such as rodents but also farm, wild, and domestic mammals [4].

Asymptomatic form of leptospirosis with fever, headache, and myalgia that can spontaneously resolve is one of clinical presentations. However, more severe cases, with sepsis and multiple organ failure, including hepatic and renal dysfunctions associated to pulmonary hemorrhage, are also reported [2].

Studies *in vivo* have been performed to reproduce the clinical disease in order to understand *Leptospira* invasion and colonization during pathogenesis due to the variability of clinical presentation of leptospirosis in humans [2].

In spite of hamsters being considered the most susceptible animal for *in vivo* studies, different select mice strains have also been used, since they are considered resistant to disease development [5–9]. It is known that selected mice strains, high and low antibody producers against complex natural antigens, show different host cellular and antibody response after *Leptospira interrogans* infection when compared with BALB/c mice at five weeks of age, probably due to immunological system competence [5, 6]. However, it is also known that mice aged one week or less are susceptible to infection when their immune system is still under development [5–7]. The importance of outbreed BALB/c mice on leptospirosis pathogenesis studies is due to their heterogeneous phenotype and also to susceptibility to be infected at one week of age as demonstrated before [5].

Cellular apoptosis, or programmed cell death, is an essential mechanism for embryonic development and host response against many infectious and non-infectious disease [10]. Apoptosis events followed by tissue injury are well documented, including in many renal diseases [11]. Caspases are a family of cysteine proteases that mediate apoptosis induced by a variety of stimuli [12]. Based on their structures and order in cell death pathways, caspases can be divided into initiators (caspase-2, −8, −9, −10 and −12) and effectors (caspase-3, −6 and −7). Two pathways, intrinsic and extrinsic death pathways, have been identified in most cases of caspase-dependent apoptosis [10]. Moreover, the effector caspase-3 can be activated by both death pathways, directly by intrinsic pathway and indirectly by extrinsic death pathway [12].

*L. interrogans* serovar Icterohaemorrhagiae has been described to invade Vero cells and induce macrophages apoptosis [13]. Besides, *in vivo* apoptosis of hepatocytes of guinea pigs infected by the same serovar has been described [14]. It has been demonstrated that *L. interrogans* induces apoptosis in J774A1 cells by activation of caspases-3 through activation of caspase-8 [15]. The role of apoptosis in the pathogenesis of leptospirosis remains unclear. Similarly, the mechanisms by which leptospirosis induces the activation of cell death needs to be elucidate [4, 16, 17].

The aim of the present study was to investigate whether apoptosis is close related to *L. interrogans* serovar Icterohaemorrhagiae by infection of young BALB/c mice at 2, 4, 7, 10, 14 and 21 days post-infection (dpi). Leptospira-induced lung, kidney and liver dysfunctions were assessed by histological parameters and anti-caspase-3 antigens were detected by immunohistochemistry assay.

## Methods

### Mice and inoculum

The animal experiments were conducted with approval from the Research Ethics Committee of São Paulo State University (UNESP). Thirty young BALB/c mice at five days of age, male and female, were divided into six groups of five mice each. For each experimental group, the same number of animals was considered as control group.

Animals were intraperitoneally injected with 1 × 10^8^ *L. interrogans* serovar Icterohaemorrhagie (strain 11437), which was previously cultured in Fletcher medium for seven days. Negative control for each group studied was injected with 1 mL of Fletcher medium alone. The *Leptospira* sample was provided by the Laboratory of Oswaldo Cruz Foundation (FIOCRUZ), maintained in semi-solid Fletcher medium and quantified according to the Faine’s technique. Mice were bled from the retro-orbital plexus at 2, 4, 7, 10, 14, and 21 dpi. During necropsy, lung, liver and kidneys were removed and fixed in 10 % neutral buffered formalin and paraffin-embedded for histological and immunohistochemistry. Tissue samples were inoculated into Fletcher medium and incubated at 28 °C to assess bacterial recovery and confirm infection by *Leptospira* spp.

### Histological

The 3 μm-thin sections from the lung, liver and kidneys collected at 2, 4, 7, 10, 14 and 21 dpi were analyzed after stained according to hematoxylin and eosin (HE) standard procedure described before [6]. The main microscopic alterations related to apoptosis were documented: adherence to the extracellular matrix and neighboring cells, chromatin condensation, intranuclear DNA fragmentation and formation of apoptotic bodies [18]. All analyses were “blind”, i.e., the researchers did not know which samples belonged to which experimental group (infected and uninfected). The images were collected under an AxioImager® A.1 light and a microscope connected to AxioCam®MRc (Carl Zeiss, Germany).

### Immunohistochemistry

For immunohistochemistry, endogenous peroxidase activity was blocked after dewaxing by incubating the sections in 2 % (v/v) hydrogen peroxidase (30 vol.) diluted in 50 % (v/v) methanol for 30 min. Pre-treatment for antigen retrieval were performed according to the specifications for primary antibody anti-caspase-3 at dilution of 1:200 in phosphate buffered solution (PBS), pH 7.2, added with 0.5 % Tween (Sigma-Aldrich, USA). Non-specific binding was blocked with 3 % (w/v) nonfat dry milk in PBS pH 7.2 for 30 min. The sections were incubated with the primary antibody for 18–22 h at 4 °C in a humidified chamber. The slides were washed in PBS and incubated with biotinylated secondary antibody and streptavidin-HRP complex (LSAB+ Kit, K0690, Dako) according to the manufacturer’s instructions.

The reaction was developed with wit 3,3′-diaminobenzidine (K3468, Dako). The slides were counterstained with Harris’s hematoxylin, dehydrated, cleared, and mounted with coverslips. Negative control sections were prepared by replacing the primary antibody with 1 % (v/v) bovine serum albumin (BSA). The tissue samples, lung, liver and kidney were examined by light microscopy. The intensity of caspase-3 labeling was scored on a four-point scale of 1–4. A score of 1 represented mild; a score of 2, moderate; 3, intense; and a score of 4 represented very intense labeling. The quantification of caspase-3 positive cells was performed by counting the number of positive cells/mm^2^ in a histological field. All analyses were “blind”, i.e., the researchers did not know which samples belonged to which experimental group (infected and uninfected).

For caspase-3 positive cells, significant differences between groups, and respective control animals, were determined by one-way ANOVA followed by Tukey’s multiple comparison test. A value of p < 0.05 was considered statistically significant. Data are expressed as mean ± standard deviation (SD). All statistical analyses were performed using Prism software (GraphPad®, USA).

### Ethics committee approval

The present study was approved by the Research Ethics Committee of São Paulo State University (FMVA- UNESP).

## Results and discussion

**Fig. 1:**
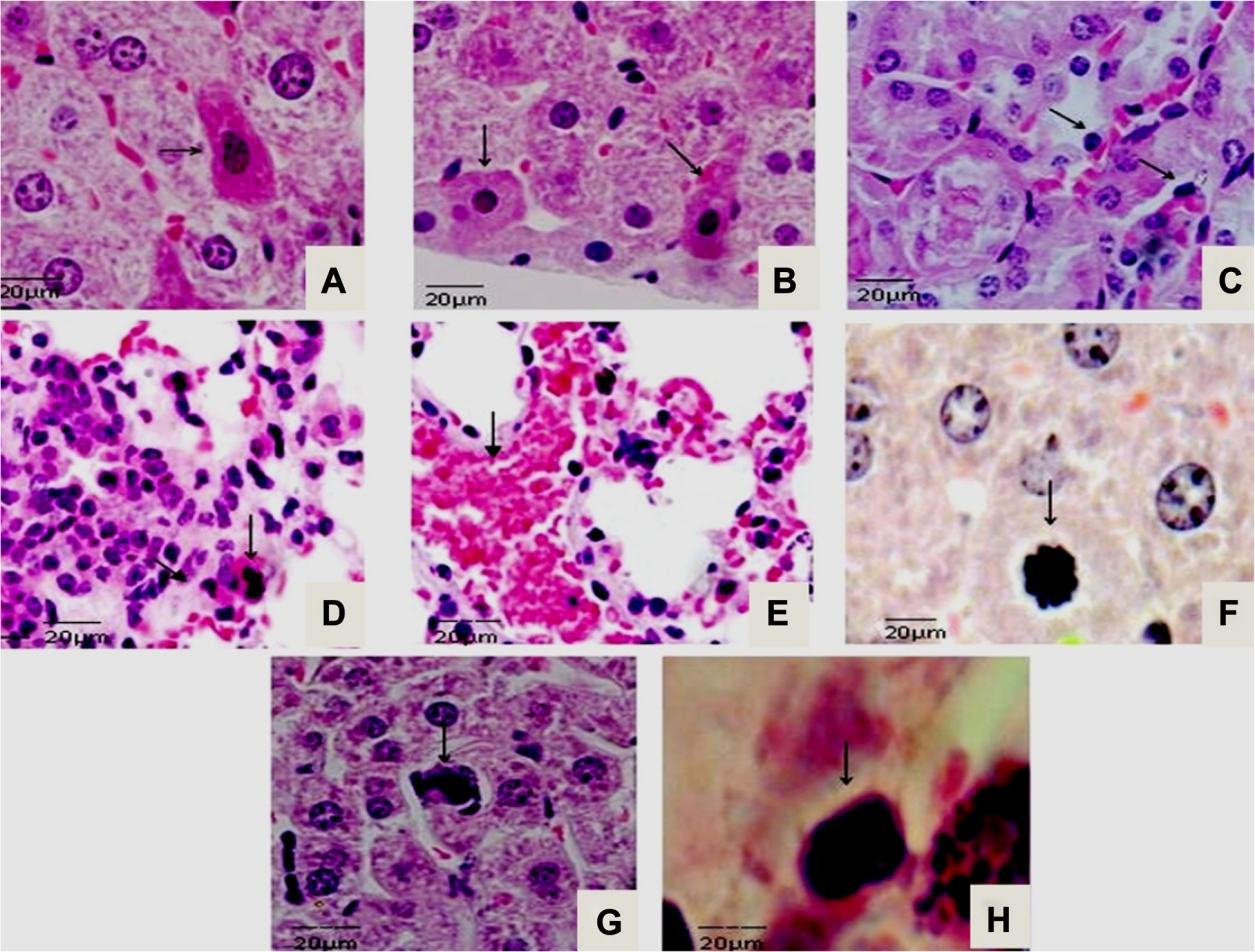
Histological lesions in BALB/c infected with *L. interrogans* serovar Icterohaemorrhage. Shown are sections of (**a** and **b**) liver, (**c**) kidneys, and (**d** and **e**) lungs. Hepatocytes showing eosinophilic cytoplasm and nuclear condensation at 14 dpi (arrows); kidneys degeneration followed by acute tubular necrosis in cortical tubules at 21 dpi (arrow); lung tissue at 14 dpi showing diffuse inflammatory cell infiltrate close to hilum region (arrow); condensation of chromatin and apoptotic bodies observed in kidney and liver sections (arrows) (n = 5 animals). Bar: 20 μm

Hepatocytes with eosinophilic cytoplasm and nuclei of chromatin fragmentation were observed at 21 dpi (Fig. 1a). At the same time, hepatocytes with cell retraction and diffuse necrosis restricted to the cortical renal tubules (Fig. 1b) and tubular necrosis near to cortical area were observed (Fig. 1c). Lung sections presenting capillary congestion and mononuclear cells infiltrates followed by polymorphonuclear cells were observed during disease progression (Fig. 1d). By 21 dpi, inflammatory cell infiltrate in pulmonary parenchyma followed by hemorrhagic areas were observed in lung sections (Fig. 1e). From 14 to 21 dpi chromatin fragmentation and apoptotic bodies were frequently observed in liver and kidney slides (Fig. 1f, g and h). The histological features from control group revealed no microscopic lesions.

**Fig. 2:**
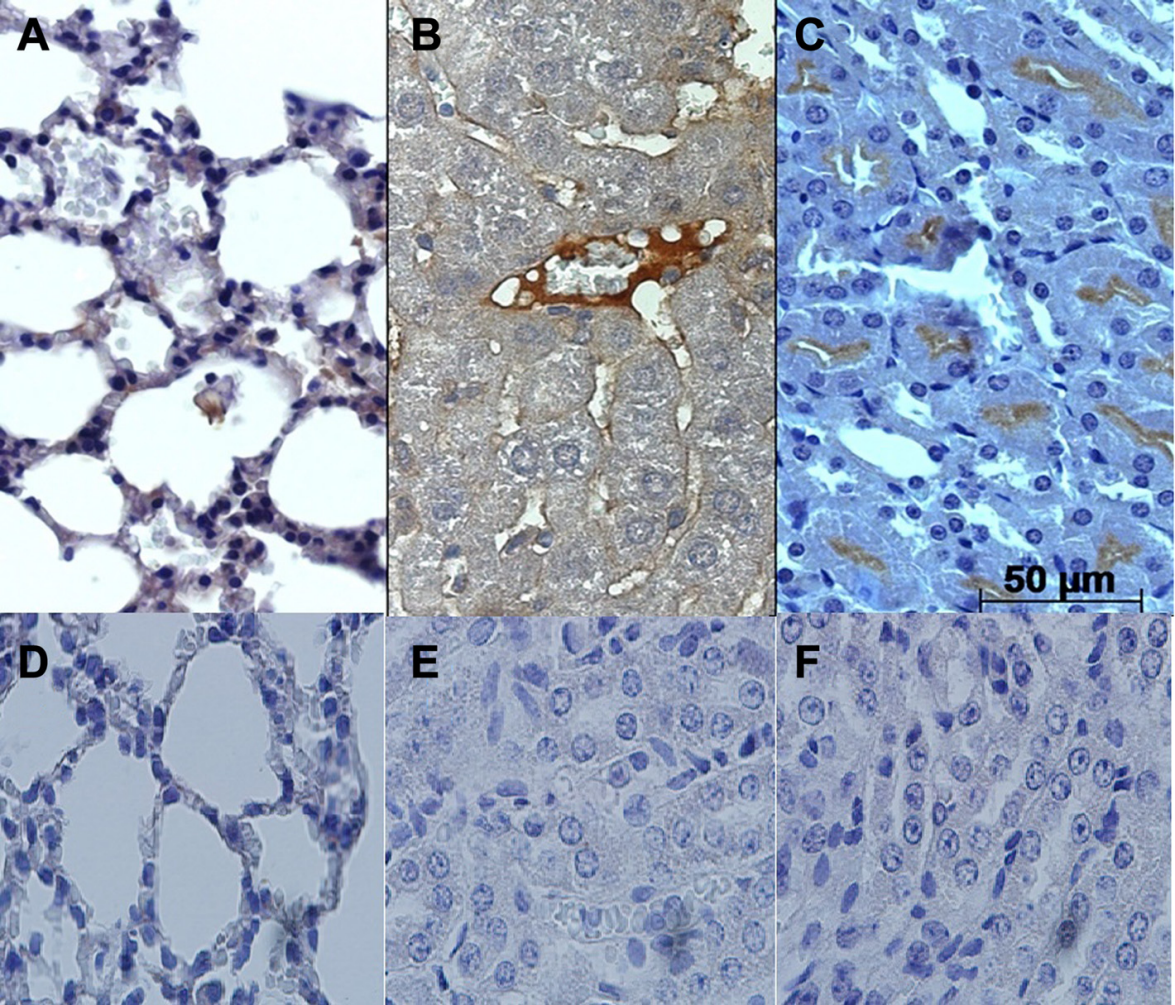
Immunodetection of caspase-3 reactive cells in BALB/c lung, liver and kidney tissue infected with *L. interrogans* serovar Icterohaemorrhage. **a** Lung samples at 14 dpi revealed positive signals in the alveolar epithelium. **b** Liver samples at 14 dpi showed centrilobular vein endothelium reactive to caspase-3 antigen. **c** In the kidney, at 21 dpi, tubular epithelial cells positively labeled for caspase-3 were observed. (**d**, **e** and **f**). Lung, liver and kidney respective controls (n = 5 animals)

**Fig. 3:**
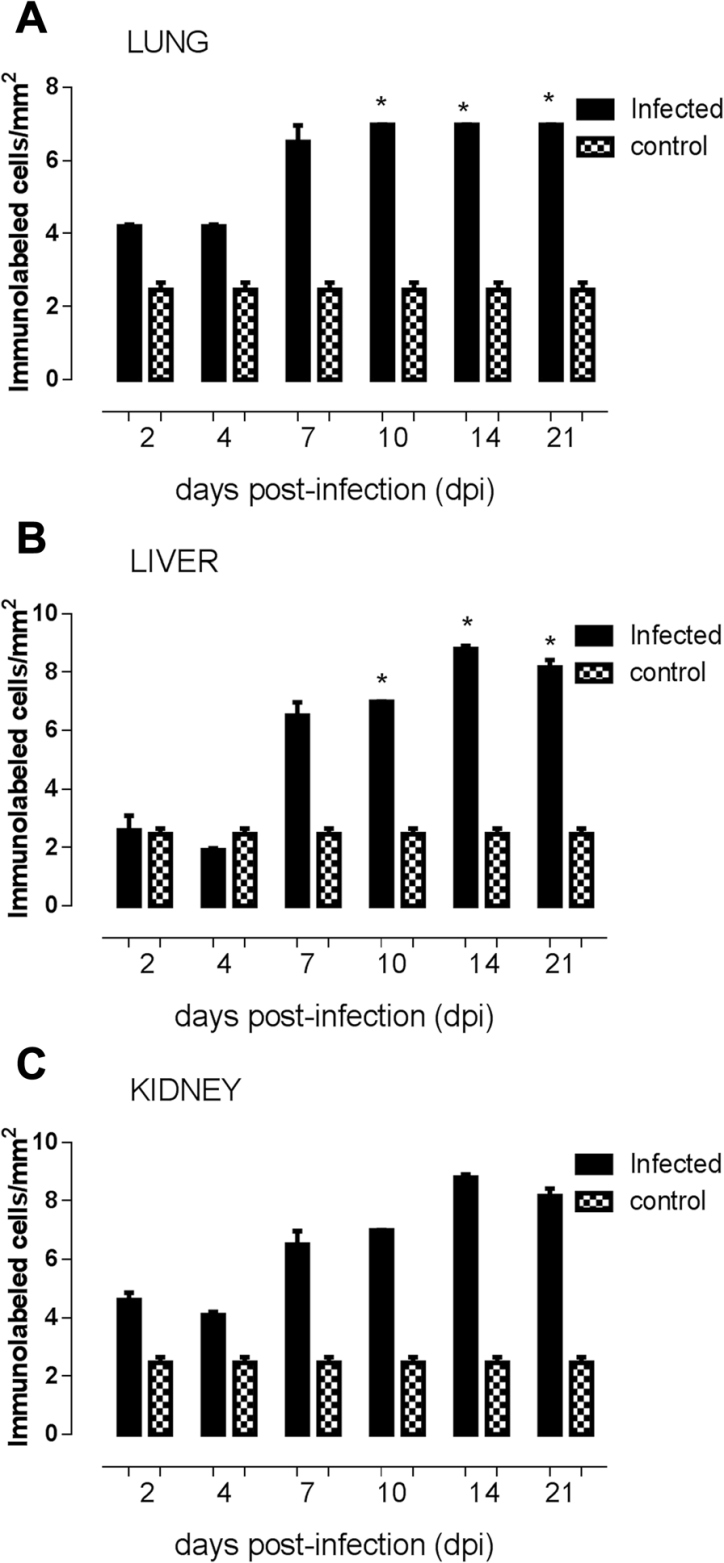
Time course of caspase-3 reactive cells quantification in in BALB/c lung, liver and kidney tissue. **a** Lung sections at 7, 10 and 14 dpi presented the highest levels of caspase-3 positive cells compared to initial dpi. **b** Number of caspase-3 reactive cells in liver sections mostly detected at 14 and 21 dpi. **c** Kidney sections showed superior number of caspase-3 reactive cells at 14 and 21 dpi, both compared to early stages of infection and to control group. Values are means ± SD (n = 5 animals). * Statistically different, p < 0.05

Caspase-3 reactive cells in the lung sections were observed in alveolar epithelium from 7 to 14 dpi (Fig. 2a). Centrilobular vein in liver sections were considered the most reactive to caspase-3 antigens from 10 to 14 dpi in liver slides (Fig. 2b). In kidney sections, the cortical region of renal tubules revealed intense reactive cells to caspase-3 antigen at all dpi (Fig. 2c). The control group is demonstrated by negative results in the lung, liver and kidney slides (Fig. 3a, b and c; black bars). At 4 dpi, the number of caspase-3 positive cells was considered significantly lower (p = 0.0497) in comparison to 7, 10 and 14 dpi, and also related to control group in lung slides (Fig. 3a). Regarding to the number of caspase-3 positive labeled cells in liver sections, a significant difference (p = 0.0069) was detected at 14 and 21 dpi when compared to control group (Fig. 3b). Analysis of kidney sections showed a higher number of caspase-3 positive labeled cells (p = 0.025) at 14 and 21 dpi, in comparison to early stages of infection and to control group (Fig. 3c).

Leptospirosis is responsible for a severe infectious disease in humans. In spite of great importance of leptospirosis for public health, the mechanism involved in the pathogenesis remains unclear [1]. Several animal models are available to study *in vivo Leptospira* infection [5–7, 9]. The hamster model is considered susceptible to develop severe fatal leptospirosis similar to what is observed in human cases [5–7]. Moreover, at early age, BALB/c mice are susceptible to the virulent strain of *Leptospira interrogans* serovar Icterohaemorrhagie, different from animals at 5 to 7 weeks of age when they can be considered resistant [5–7].

Pathological changes observed in this study demonstrated the BALB/c susceptibility to *Leptospira interrogans* serovar Icterohaemorrhagie infection, especially after seven days after infection. Herein, pulmonary lesions, characterized as edema, capillary hemorrhage and hemoptysis are manifestations of severe leptospirosis in humans, confirming the severe disease developed by young BALB/c mice model [7, 19, 20]. Same histological lesions have been reported in hamsters consistent with our results and contrary to what have been demonstrated in resistant animals [7, 9, 20, 21].

The mechanism by which leptospires cause host tissue damage or, conversely, the pathway used by infected hosts to avoid organ failure is still unclear. Few studies have focused in the liver histological features of animal models of leptospirosis. Inflammation of the portal tract in the liver of hamsters with numerous inflammatory elements, ending in a massive necrosis, has been described [7, 9]. In this study, liver histological changes were observed with an increase at late periods of observation. These achievements are in agreement with previous studies in which leptospirosis is well characterized as a biphasic illness [4]. The acute phase is represented by a great number of circulating bacterial forms followed by clearance/initial apoptosis spread in target organs such as lung, liver and kidney [16]. The clearance phase of *Leptospira* infection *in vivo* has been associated with antibody increase and high levels of inflammatory cytokines, such as TNF-α and IL-6 [5–7, 9, 22, 23]. Both kidney and liver histological analysis show an increase of chromatin fragmentation and apoptotic bodies at late periods of observation after *L. interrogans* infection.

Acute renal failure in a hamster model of *Leptospira* infection has been previously observed, which exhibited the same histological damages observed in kidney tissue in the present work [5–7]. In contrast, resistant BALB/c mice infected with a virulent strain of *L. interrogans* serovar Canicola revealed less histological lesions when compared to *L. interrogans* serovar Icterohaemorrhagie in this study [5, 8]. Previous studies have demonstrated that an early regulation of chemokines and proinflammatory cytokines in mouse kidneys might be associated with limited pathological lesions; however, no study measured these factors in BALB/c at early age [5–7, 9, 22, 23].

Apoptosis of host cells plays an important role in modulating the pathogenesis of many infectious diseases. It has been reported that *L. interrogans* induces apoptosis in macrophages *in vitro* and hepatocytes *in vivo* [13, 14, 24, 25]. This study represents the first description of apoptotic features in lung, liver and kidney obtained from BALB/c mice infected with *L. interrogans* serovar Icterohaemorrhagie. Caspase-3, serving as an executor caspase, plays a pivotal role in the apoptosis induced by various pathogens. In addition, *in vitro* studies have demonstrated that activation of caspase-3 is not the only mechanism leading to *L. interrogans*-induced apoptosis [24]. However, once caspase-3 is activated, it will be very difficult to reverse the programmed cell death [10].

The present findings demonstrated an increase of reactive caspase-3 cells distributed in lung, liver and kidney sections at after seven days post-infection, contrary to studies performed *in vitro*, whereas reactive cells were detected at early stages after *L. interrogans* infection [12, 17, 24, 26]. It is important to emphasize that *in vivo* infections reproduce the clinical signs according to the bacterial spread into the body, contrary to *in vitro* models, whereas intrinsic pathogen-cell signaling is specifically characterized. Both models have a great of importance; however, their differences must be taken into account when comparisons are made.

The results presented here revealed an increase of reactive caspase-3 cells in the lung, liver and kidney at late periods after infection with *L. interrogans*. These results corroborate previously described histological changes related to apoptosis; however, they contradict other *in vitro* studies [13–15, 18, 19, 24]. Further studies should be conducted to determine the factors involved in activation or inhibition of apoptosis pathways in *L. interrogans* infection. Blocking these keys responsible for self-destructive reactions would minimize tissue injury and inflammatory response.

## Conclusions

Our results suggest that infection with *L. interrogans* induce an activation of apoptosis mediated by caspase-3 dependent pathways in later phases of the infectious process in the kidney, liver and lung tissues.
